# Calling by Domestic Piglets during Simulated Crushing and Isolation: A Signal of Need?

**DOI:** 10.1371/journal.pone.0083529

**Published:** 2013-12-13

**Authors:** Gudrun Illmann, Kurt Hammerschmidt, Marek Špinka, Céline Tallet

**Affiliations:** 1 Department of Ethology, Institute of Animal Science, Prague, Czech Republic; 2 Cognitive Ethology Laboratory, German Primate Center, Göttingen, Germany; 3 INRA, UMR1348 PEGASE, Saint-Gilles, France; 4 Agrocampus Rennes, UMR1348 PEGASE, Rennes, France; CNR, Italy

## Abstract

This study examined whether piglet distress vocalizations vary with age, body weight and health status, according to the predictions of the honest signalling of need evolutionary model. Vocalizations were recorded during manual squeezing (a simulation of being crushed by mother sow) and during isolation on Days 1 and 7 after birth in piglets from 15 litters. We predicted that during squeezing, younger, lighter and sick piglets would call more intensely because they are in higher risk of dying during crushing and therefore they benefit more from the sow’s reaction to intensive vocalization. For isolation, we predicted that lighter and younger piglets would call more because they are more vulnerable to adverse effects of the separation. Calls were analyzed in their time and frequency domain. The rate of calling, call duration, proportion of high-pitched calls and eight acoustic parameters characterizing frequency distribution and tonality were used as indicators of acoustic signalling intensity. Piglets that experienced “squeezing” on Day 1 produced more intense acoustic distress signalling than on Day 7. Lighter piglets called more during squeezing than heavier piglets. Health status did not significantly affect any of the indicators of intensity of vocalization during squeezing. In isolation, none of the parameters of vocalization intensity were affected either by the age or by the weight of the piglets. In summary, the model of honest signalling of need was confirmed in the squeezed situation, but not in the isolation situation.

## Introduction

### Offspring distress vocalizations

Acoustic communication is a prominent element of parent-offspring interaction in many vertebrate species [1]. An important part of this communication can be distress vocalizations through which the progeny solicit parents’ help when their fitness is at stake, e.g. when they are isolated, in pain, injured or under predator attack [2-5]. Long, tonal calls are often, but not always used by vertebrate infants as distress vocalizations [6]. The variation in call rate and in the acoustic properties of these calls such as duration, fundamental frequency and frequency distribution can convey information about the state of the offspring and/or the gravity or severity of the situation. The main evolutionary model that has been proposed to explain how the variation in offspring signalling can convey reliable information to parents is the honest signalling of need model.

### Honest signalling of need

In a system of honest signalling of need between parents and offspring, the progeny addresses the parents with signals that rise in rate and/or intensity with increasing need of the offspring and parents provide more resources in response to increased solicitation [7,8]. In the classical model of parent-offspring honest signalling, the reliability of the relationships between the degree of need, the intensity of signalling and the level of parental care is maintained by different cost-benefit balances in the more and the less needy piglets. The costs of signalling are similar for both the more and less needy offspring, but the benefit from the increased parental input is higher for the more needy progeny [8-10]. In spite of the potential for parent-offspring conflict, the signalling system is stable and provides the parent with accurate information about the resource needs of its young [9]. The stability is based on the costs of the signalling and on the fact that the parent, the signalling offspring and the current and future siblings of the offspring are genetically related. There is extensive empirical support for this model, especially in the context of progeny food provisioning, in a variety of species of birds and mammals [11-14]. The term “need” is often used as a synonym of short-term variation of offspring condition such as satiation level or the degree of thermoregulatory challenge [2,12,15]. For instance, Lotem [16] interpreted the term ‘need’ as „the marginal benefit from obtaining extra food“. However, the model also applies to situations outside the food provisioning context. Offspring signalling may influence the amount or probability of various types of parental care, including protection against danger or pain alleviation. Also, the model need not be restricted to short-term changes in offspring condition. For instance, Weary and Fraser [14] considered that the more needy piglets are both those that have not sucked milk recently (a short-term change in condition) and those that were of low body weight or grew slowly (a long-term variation in condition) and Lotem [16] took the same approach in barn swallows. Weary and colleagues [15,17] also examined the effect of age at weaning on piglet vocalisations within a similar framework. They interpreted higher intensity of calling in earlier weaned piglets as a reliable signal of their lack of adaptation to independent life, i.e. of their need to acquire maternal care. If need is defined as the marginal benefit from receiving an additional portion of maternal care, then it is appropriate to analyze age as yet another type of condition that affects the need of a dependent offspring. The original model by Godfray [7] explicitly covers any type of maternal care through which the offspring viability is affected. The model predicts a negative relationship between the offspring short-term or long-term condition and its signalling level.

In this paper, we apply the honest signalling of need model to piglet distress vocalisations emitted in two potentially dangerous situations during the first week of life. We focused on three long-term aspects of the piglet condition: age, body weight and health status.

### Distress vocalizations of piglets as related to age, weight and health status

Infant piglets of both wild and domestic pigs (*Sus scrofa*) are highly vocal [18,19]. They emit distress vocalizations in situations such as when they are in pain [20], hunger [14], isolation [21] and when being overlain accidentally by their mother (“crushed” in animal science jargon; here we use the term “squeezed”). We introduce here the squeezed and isolation calls (S-calls and I-calls, respectively) in more detail as these are the two types of call examined in this study.

When a piglet gets trapped under the body of the sow when she rolls over or lies down [22], it starts screaming immediately. The screams are typically long, high-pitched calls [23]. The sow usually, but not always, reacts to the screams by standing up [24,25], a behaviour that normally saves the life of the piglet [22]. The function of the S-calls is to solicit maternal care in the form of body movement reaction and the benefit for the piglet is a decreased probability of injury or even death. Fatal piglet squeezing is mostly limited to the first 3 days post-partum [26]. Being squeezed is not usually a life threatening event for older piglets. Thus, the need of the piglet for the maternal reaction is highest during the first three days and therefore we predict that the vocal signalling through the S-calls will decline in intensity after Day 3. The S-calls may also vary with the body weight of the piglets. Piglets of low weight are more likely to die by being crushed [27] and this may be not only due to higher probability of getting trapped under the sow but also due to the lower survival chance once trapped under the sow. These two aspects have not been yet properly separated in published studies [26,28]. Nevertheless, the probability of dying increases steeply with the amount of time the piglets is trapped under the sow [29]. Therefore it seems plausible that lighter piglets with less muscle power will be less successful in freeing themselves in time before suffocating. If so, then lighter piglets should react with more intense S-calls when they are squeezed. The same may apply to sick piglets. Although no study - to our knowledge - investigated how health status affects piglet’s mortality if crushed [27], there are good reasons to assume that sick piglets are less able to free themselves from under the sows. For instance, piglets with diarrhea may be weaker due to dehydration and hypoglycaemia [30]. Other pathologic conditions cause anemia [31] that may also compromise the ability of piglets to exert forceful and sustained whole-body muscular activity due to reduced oxygen supplementation of the muscles. 

Piglets also call when isolated from the sow, typically through short, low-pitched calls also described as grunts [32]. Weary and Fraser [14] investigated piglets put into isolation at 10 days of age and found that hungry piglets, slow-growing piglets and piglets exposed to colder environments called more often and used longer, louder and higher pitched calls than satiated piglets, fast-growing piglets and piglets in warm environments, respectively. These findings indicated that piglets in poorer short-term or longer-term body condition and therefore in greater need of the sow’s care call more intensely compared to those in lesser need. Also, sows responded more towards needy piglets in playback experiments [15]. An age effect has been shown for I-calls in 1-4 weeks old piglets. The call rate was lower for older piglets, especially for high frequency calls [33]. Functionally, this is probably because the older much heavier piglets are more likely to survive a prolonged period of isolation due to higher energy reserves and also due to their ability to consume solid food. Neither the development of isolation calls nor the weight effect on isolation calls of piglets within the first week of life, have been investigated. We predict, based on the results of Weary et al [33] who studied older piglets, and in agreement with the honest signalling of need model, that very young piglets and physically smaller piglets will vocalize more intensely than their stronger litter mates. We base this prediction on the fact that the ability of a 1 day old piglet to survive a prolonged separation is lower than that of a 7 day old piglet due to lower body reserves and higher critical ambient temperature [27,34]. Therefore the risk of hypothermia and starvation (and the subsequently increased danger of crushing and death) is much higher at Day 1 than at Day 7 [26] and consequently every separation from the sow at this early age can be very dangerous.

The aim of the study was to determine whether the intensity of piglet distress vocalization is higher in piglets that are more vulnerable in three dimensions of their condition, i.e. that are younger, lighter or sick. Rate of calling, call duration, proportion of high-pitched calls and eight acoustic parameters characterizing the frequency distribution and tonality were used as indicators of acoustic signalling intensity. Two predictions (Prediction 1 and Prediction 2) for the squeezed situation and one prediction for the isolation situation (Prediction 3) were tested. 

Prediction 1. In the squeezed situation, one day old piglets and lighter piglets will vocalize more intensely than seven days old piglets and heavier piglets because they are in higher danger of dying in this situation.

Prediction 2. In the squeezed situation, sick piglets will vocalize more intensely than healthy piglets because they are more at risk due to their weakened physical condition. 

Prediction 3. In isolation, one day old piglets and lighter piglets will call more intensely, because it is more dangerous for them to remain separated from their mother than it is for seven days old piglets and heavier piglets.

## Materials and Methods

### Ethics statement

This study received approval for animal use and care from the Institutional Animal Care and Use Committee of the Institute of Animal Science and was conducted in accordance with Czech Central Committee for Protection of Animals number 44248/2007-17210.

### Subjects and study site

The experiment was carried out in 2008 and 2009 at the Institute of Animal Science located in Prague, Czech Republic. We used 15 Large White Landrace sows in this study. The sows farrowed in pens with concrete floors and straw bedding measuring 2.3 m x 2.0 m within a room containing 14 such pens. Each pen was equipped with a ‘walk around’ ellipsoid farrowing crate (2.3 m x 1.4 m) with a small partition in its centre. The crate allowed the sow to walk around in one direction, but not to turn around or to reach the piglets’ creep area (2.3 m x 0.6 m) located in the corner of each pen. Supplementary heat from a warm plate in the creep area was provided during the first 2 weeks. The sows were fed a standard lactation diet twice a day. Water was continuously available from one nipple for the sow and another for the piglets.

### Data collection

Piglets from 15 litters were used for the call recordings. Litter size was 12.1 ± 2.1 (mean ±SD), there were 43. 7 % males among the piglets included in the experiments. Piglets were weighed, marked with a number on the back and the rectal temperature was measured on Day 1 and Day 7 post-partum (hereafter: pp). The mean body weight on Day 1 was 1548.7 g ± 414 (mean ±SD) and on Day 7 it was 2565.3 g ± 804 (mean ±SD). The health status of every piglet was judged for 5 different indicators of disease. Each piglet was observed individually and we noted the presence or absence of each indicator, namely quality of respiration (i.e. presence of coughing, sneezing, and labored breathing), enteric disease (diarrhea), neurological disease (muscle tremor, paddling movements), signs of apathy (animal not moving or reacting to our presence) and skin disease (dermatitis, un-colored skin indicating anemia). When at least one indicator of disease was present (e.g. diarrhea), the piglet was judged as 'sick'. Otherwise, piglets without any distinctive sign of disease were judged as ‘healthy’. Based on these criteria, we identified 14 sick piglets in our sample. All of them had just one indicator of disease: respiratory disease (1 piglet), enteric disease (10 piglets) and skin disease (3 piglets).

The two lightest healthy and two heaviest healthy piglets were chosen for each of the 15 litters on Day 1. From these piglets, one heavy and one light piglet (randomly chosen) from each litter was selected for recording of the vocalization during manual squeezing; the other pair of light and heavy piglets was used for recording the vocalization during isolation on Day 1. On Day 7 pp, the two lightest and two heaviest piglets were identified once more according to their current weight and used for testing. If these were the same piglets as on Day 1, then always the piglet that had been tested during simulated crushing on Day 1 was tested during isolation on Day 7 and vice versa. Thus, no piglet was tested twice in the same situation and age was a between-subject factor in the analysis.

Additionally, we tested the effect of sickness on piglets of same age and similar weight. In every litter with a sick piglet, this piglet and another healthy piglet with a similar body weight were tested. Because of the low number of sick piglets, we tested a sick piglet and another healthy piglet only during periods of manual squeezing but not during isolation.

### Recording of S-calls

S-calls were recorded during a manual squeezing situation lasting 30 sec, carried out in a separate room on Day 1 and Day 7 respectively for the lightest healthy and heaviest healthy piglet and as well for a sick piglet and a litter mate with a similar body weight. 

A piglet was held around the chest and restrained in a lateral position on a weigh scale with the experimenter applying a controlled pressure of about 5 kg. This method was used previously [25] and validated, i.e. the sows reacted in the same proportion to simulated crushing calls and real crushing calls. A microphone was held 1.0 m away from the snout of the piglet. All recordings were carried out using a microphone (Sennheiser ME 67) connected to a digital recorder (Marrantz PMD 660, sampling rate of 44.1 KHz, 16-bit). We identified calls visually using spectrograms created by Avisoft SAS-Lab Pro (Specht). Each call was saved into separate digital file.

### Recording of I-calls

I-calls were recorded on Day 1 and Day 7 pp respectively. The piglet was separated from its mother and littermates immediately after a successful nursing, i.e. a nursing with milk ejection. This procedure ensured that hunger did not influence the piglet vocalization [14]. The piglet was brought into a separate room and put into a box (0.5 x 0.5 x 0.5 m). A microphone was positioned 10 cm below the top of the box such that the distance between the piglet’s mouth and microphone was approximately 0.5 m. After the piglet was placed in the enclosure, the recording, using the same equipment as for S-calls, was started and the piglet was recorded for 10 min and simultaneously video was recorded. The same technique was used as for recording the S-calls.

The latency of the first vocalization was noted. All I-calls occurring during the first 20 sec of the 2^nd^, 4^th^, 8^th^ and 10^th^ min were sampled. The calls were visually inspected and all calls that were not disturbed by background noise were sampled. To obtain the optimal frequency resolution, we reduced the sample frequency (as described below). 

### Acoustic analysis

To analyze the acoustic structure of S-calls we cut out single calls and conducted a fast Fourier transform (1024-pt FFT; time step: 2.9 ms; frequency range: 22 kHz; frequency resolution: app. 45 Hz) using Avisoft SASLab Pro 4.3 (R. Specht, Berlin, Germany). Because the I-calls of piglets have a lower and smaller frequency range [33] we reduced the sampling frequency to 11 kHz to have an appropriate range to estimate energy distribution and fundamental frequency (F0). Afterwards we used the same procedure to produce frequency spectra (1024-pt FFT; time step: 2.9 ms; frequency range: 5.5 kHz; frequency resolution: app. 11 Hz).

**Figure 1:**
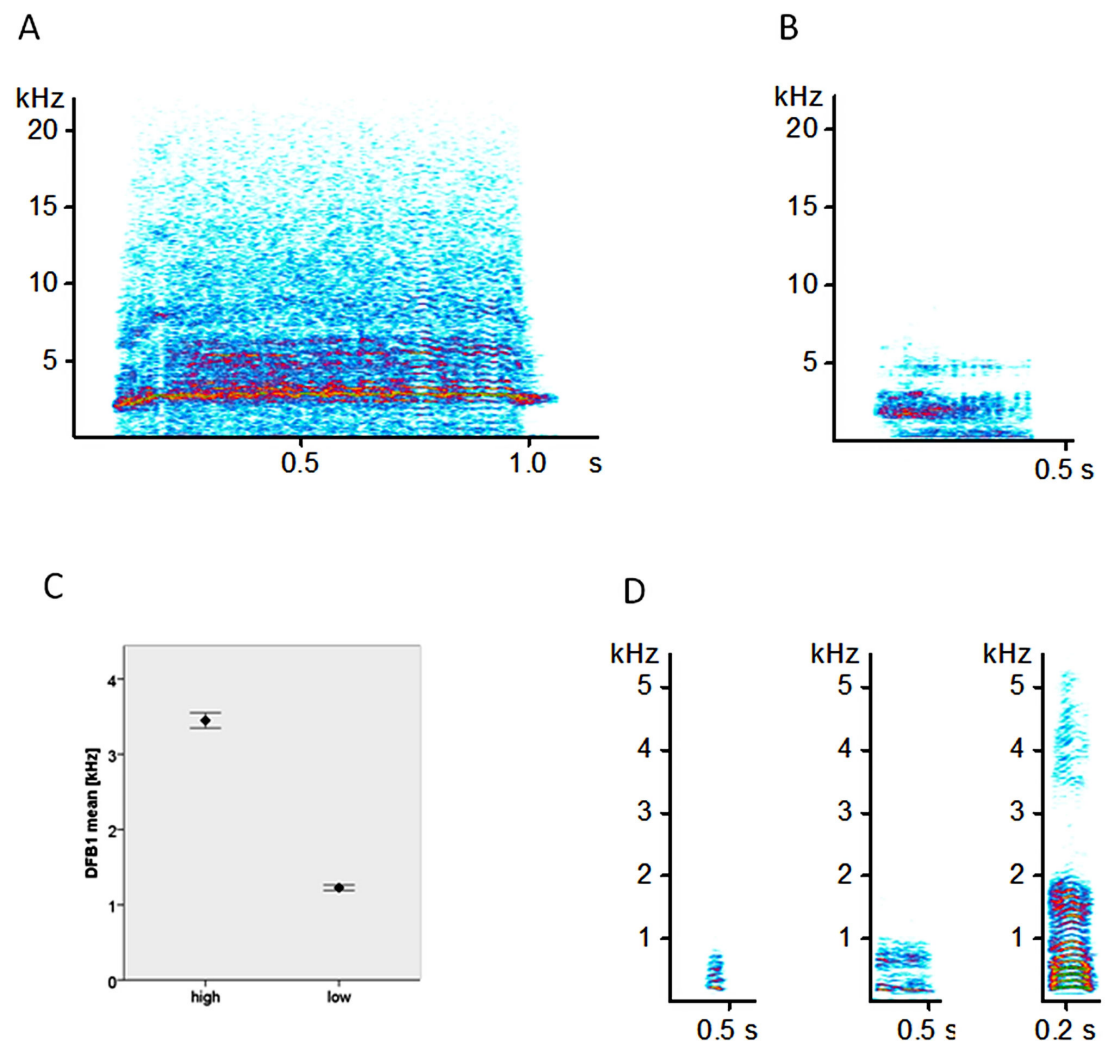
Spectrogram of a typical high-pitched S-call (A), spectrogram of a typical low- pitched S-call (B), Differences in the 1st dominant frequency band, the lowest dominant frequency of call, between high and low-pitched calls (C), Three spectrograms of typical I-calls (D).

We used a custom software program (LMA 2009, available on request from KH), to calculate acoustic parameters best suited to characterize the structure of S-calls and I-calls [35]. As S-calls (Figure 1A, 1B) and I-calls (Figure 1D) fundamentally differ in their tonality we used a different set of acoustic parameters to describe both call types. A description of the acoustic parameters used in the analysis is given in Table 1. To describe the distribution of frequency amplitudes within the spectrum we estimated their statistical distribution. To calculate the distribution of frequency amplitudes (hereafter DFA), we first determined the overall amplitude. Subsequently, we calculated the frequency at which the distribution of the amplitude in the frequency spectrum, reaches the first, second and third quartile of the total amplitude. Secondly, we calculated the dominant frequency bands (DFB). The dominant frequency bands are characterized by amplitudes that exceed a given threshold in a consecutive number of frequency bins. The numbers of the dominant frequency bands count from the lowest frequency up; the first DFB is not necessarily the DFB with the highest amplitude. In tonal parts of vocalizations the first DFB corresponds to the fundamental frequency. We also specified the modulation of the first DFB and the peak frequency, the frequency with the highest amplitude in a certain time segment. All these calculations were done for each time segment.

**Table 1:** Description of call parameters used in the statistical analysis of distress vocalization during squeezing (S-calls) and during isolation (I-calls).

**Acoustic parameters**	**Description**	**Included in the statistical analysis**	
		**S-calls**	**I-calls**
Duration [ms]	Time between onset and offset of call	Yes	Yes
DFA2 mean [Hz]	Mean value of the frequency at which the second quartile of global energy is reached across all time segments	Yes	Yes
DFA3 mean [Hz]	Mean value of the frequency at which the third quartile of global energy is reached across all time segments	Yes	Yes
PF max [Hz]	Maximum frequency of the frequencies with the highest amplitude across all time segments	Yes	Yes
PF mean [Hz]	Mean of the frequencies with the highest amplitude across all time segments	Yes	Yes
DFB1 start[Hz]	Start value of the first frequency in the call that contains more energy than a particular threshold across all time segments	Yes	Yes
DFB1 mean [Hz]	Mean value of the first frequency in the call that contains more energy than a particular threshold across all time segments	Yes	Yes
DFB1 mod [Hz]	Mean difference between original DFB course and an average course	Yes	No
F0 mean [Hz]	Mean value of the fundamental frequency across tonal time segments	No	Yes
Noisy parts [%]	Percent of time segments in which no harmonic structure can be detected	No	Yes

Because I-calls are mainly tonal vocalizations (Figure 1D) we estimated the amount of tonality and the fundamental frequency (F0). To estimate these parameters we calculated the autocorrelation function of every time segment of a given I-call. Depending on the number and periodicity of the peaks of the autocorrelation function it is possible to classify a time segment as noisy - no peaks could be detected, complex - some peaks could be detected but they were not periodic, or tonal - peaks were periodic. To test whether the produced calls (S-calls and I-calls) had a heterogeneous distribution we used a two-step cluster procedure (SPSS 19) to test for different call clusters. We used the log-likelihood distance measure to establish different vocal cluster (up to 15 clusters) and the Schwarz-Bayesian information criterion (BIC) to decide which cluster solution showed the best fit. The procedure found a two cluster solution as the best division of S-calls. This result was supported by the second independent cluster analysis of S-calls of healthy and sick piglets, which also revealed the two cluster solution as the ‘best’ solution. For I-calls the two-step cluster procedure found improving cluster solutions, confirming that the one cluster solution is the best description for the existing variation of I-calls.

### Statistical analysis

To assess which factors affected the acoustic quality of S-calls we used linear mixed models (SPSS 19), with age, weight (i.e. actual body weight on the day of testing, a continuous effect) and the interaction between age and weight as between-subject fixed factors. Piglet identity (subject) was included as a random factor because several calls per piglet were used in the analysis. SSType1 was applied with age as the first factor. In the statistical analysis we included sex of the piglet as a categorical factor. However, this factor was removed from all models based on its non-significant effect in all models. In total we had 59 piglets and 1430 S-calls in the analysis. For the comparison of healthy and sick animals we carried out a separate analysis and added health status as an additional fixed factor. In this analysis we had 28 piglets with 776 S-calls. Factors affecting I-calls quality were also tested with a linear mixed model (SPSS 19), with age, weight, duration of isolation (after 2-4 min and 8-10 minutes), and the interaction between age and weight as fixed factor and subject as random factor. In total we analyzed 3422 calls from 46 piglets. In the cases when we repeatedly tested against the same global hypothesis we used Simes correction to adjust for multiple testing*.*

## Results

### Effect of age, weight and health on S-calls

For testing of Prediction 1, 891 S-calls were of sufficient quality for analysis. The two-step cluster analysis categorized 555 (62 %) calls as high pitched and 336 (38 %) as low- pitched calls (Figure 1A, 1B, 1C). 

**Figure 2:**
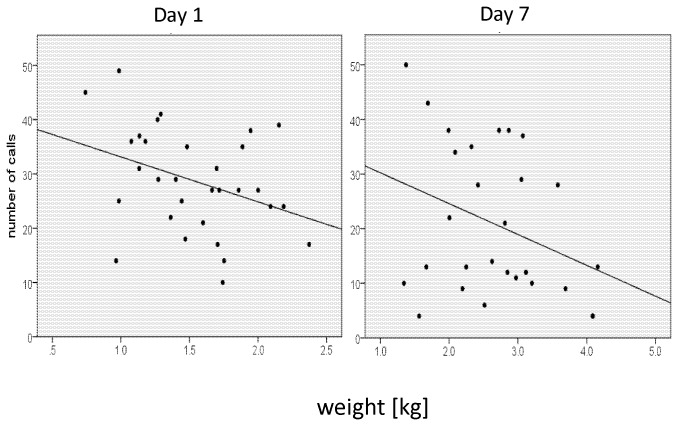
Number of S-calls (both high and low-pitched calls) on Day 1 and Day 7 as dependent on piglet weight.

Prediction 1 was strongly supported for the age effect. In the squeezed situation, piglets on Day 1 produced more intense acoustic distress signalling calls than on Day 7 (Table 2). On Day 1, piglets called more often (Figure 2; F_1, 55_=7.1, P=0.01, Day 1: 28.7 ± 1.7 vs. Day 7: 20.9 ± 2.6, mean ± SEM), and had a higher proportion of high-pitched calls (F_1, 55_=14.8, P= 0.0001, Day 1: 71.6 % ± 4.9, Day 7: 45.3% ± 4.9) than Day 7. The high-pitched calls were significantly longer (P=0.013) and had a significant higher dominant frequency band (DFB1 start and DFB1 mean, P<0.01, Table 2) on Day 1 than on Day 7. 

**Table 2:** Differences in S-calls in relation to weight and age for high-pitched calls.

**Acoustic parameters**	**Weight**	**Age**	**Day 1**	**Day 7**
	**P value**	**P value**	**Mean ± SEM**	**Mean ± SEM**
DFA2 mean [kHz]	0.569	0.73	8.9 ± 0.3	8.9 ± 0.4
DFA3 mean [kHz]	0.517	0.635	15.3 ± 0.4	15.2 ± 0.6
PF max [kHz]	0.974	0.608	11.1 ± 0.6	11.5 ± 0.8
PF mean [kHz]	0.569	0.212	6.3 ± 0.3	5.6 ± 0.5
DFB1 start[kHz]	0.974	**0.008**	**2.5 ± 0.2**	**1.8 ± 0.3**
DFB1 mean [kHz]	0.517	**0.008**	**3.9 ± 0.2**	**2.9 ± 0.3**
DFB1 mod [kHz]	0.569	0.635	0.5 ± 0.04	0.5 ± 0.06
Duration [ms]	0.517	**0.013**	**876 ± 66.1**	**536 ± 95.8**

Significant differences are in bold. P-values show the adjusted values after Simes correction for multiple testing.

Prediction 1 was partly supported for the weight effect. Lighter piglets called significantly more often than heavier piglets (Figure 2, F_1,55_=7.1, P=0.011), in agreement with Prediction 1. However, none of the other parameters of vocal signalling intensity were affected by weight (all p’s > 0.05, Table 2). 

There was a significant interaction between age and weight (F_1, 53_=4.3, P<0.05) but the subsequent separate tests revealed no significant difference in relation to weight (Day 1: F_1, 28_=2.7, P=0.11; Day 7: F_1, 25_=1.6, P=0.21). None of the other interactions between age and weight was significant. 

 For testing of Prediction 2, a total of 764 S-calls of healthy and sick piglets were of sufficient quality for analysis. The two-step cluster analysis categorized 489 (64%) calls as high-pitched and 275 (36 %) as low-pitched calls. Health status did not significantly affect any of the indicators of vocalization intensity (all p’s > 0.05). 

### Effect of age, weight and isolation time on I-calls

In the isolation situation, none of the parameters of vocalization intensity were affected either by age or by weight (Prediction 3) of the piglets (all p’s > 0.05, Table 3). We also found no significant interactions between age and weight. The calling intensity decreased in four out of nine acoustic parameters between the 2-4 min of isolation and 8-10 min of isolation. Figure 1D displays typical examples of isolation calls emitted after 2-4 min of isolation and 8-10 min of isolation.

**Table 3:** Differences in I-calls in relation to weight, age and calling duration.

**Acoustic parameters**	**Time**		**Time**	**Weight**	**Age**
		**2-4 min**	**8-10 min**	**P values**	
Duration [ms]	**259.4 ± 11.1**	**242.8 ± 12**	**0.009**	0.998	0.618
DFA2 mean [Hz]	**752 ± 41.6**	**662.7 ± 46.5**	**0.009**	0.941	0.4121
DFA3 mean [Hz]	**1212 ± 72.3**	**1025.6 ± 80**	**0.000**	0.941	0.121
PF max [Hz]	**971 ± 68.7**	**768.2 ± 78.5**	**0.000**	0.941	0.245
PF mean [Hz]	454.6 ± 33.4	397.5 ± 37.9	0.077	0.941	0.245
DFB1 start [Hz]	244.2 ± 15.1	247.3 ± 17.1	0.663	0.941	0.618
DFB1 mean [Hz]	251.9 ± 13.2	237.1 ± 15.3	0.383	0.941	0.335
F0 mean [Hz]	148 ± 7.9	146 ± 9.2	0.858	0.941	0.618
Noisy parts [%]	65.9 3	67.1 3.2	0.383	0.941	0.618

Significant differences are in bold. P-values show the adjusted values after Simes correction for multiple testing. Means (± SEM) are estimated marginal means.

## Discussion

### The squeezed calls – age and weight effects

The age of the piglets had by far the strongest influence on the distress calls in the squeezed situation, thus clearly confirming Prediction 1. The calling by the younger piglets was more intense in several aspects, including calling rate, the proportion of high-pitched calls and the longer duration and higher acoustic frequency of these calls. Thus, mother sows that hear the calls receive more powerful action-soliciting signals from the one-day old progeny than from the one-week old piglets. 

The most probable interpretation is that the “squeezed” situation was perceived by the older piglets as much less threatening than that by the one day old piglets. Therefore the older piglets were in less urgent need of help from the mother and consequently they signalled less intensely. The squeezed situation into which the piglets were experimentally put could not, for obvious ethical and welfare reasons, be permitted to hurt or injure the piglets. The pressure applied during the restraint (5 kg) was by no means comparable to the real crushing pressure of an adult sow whose body mass is in the order of hundreds of kilograms. However, mother sows tend to lie down rather slowly and carefully [36,37]. Therefore our experimental situation probably corresponds well with the initial moments of trapping when the pressure is not so strong. In this situation, stronger piglets may be able to release themselves. However, smaller and less movement-proficient piglets may need to rely on the sow interrupting the lying-down movement to escape crushing. Therefore, the older piglets with their more advanced body condition probably did not perceive the situation as a life-threatening emergency. Indeed, less than half of the squeezed vocalizations emitted by older piglets were the high-pitched extreme-distress calls, whereas the majority was shorter low-pitched vocalizations that are rather typical of contact or low distress calls [19]. In other situations, for example during castration, piglets produced almost 90% high-pitched calls, which indicate a more ‘threatening’ situation compared to manual squeezing [20,38]. One-week old piglets almost never get fatally crushed [39] and therefore the restraint does not announce a grave danger for them. In contrast, up to 10% of all piglets die before Day 3, many of them by crushing [28,39]. For the very young piglets, the combination of an inability to move and moderate pressure may signal a more real and serious danger of being crushed by the sow. Sows tend to respond to S-calls by changing posture [25], presumably so as to release the trapped piglet. A piglet usually survives if the sow reacts in less than 1 min to the calls of a trapped piglet [22]. Younger piglets might benefit more from her response, because they are in higher risk of being injured. Thus our finding that the very young pigs vocalized much more intensely than the older piglets is in agreement with the signalling of need model [7,8]. The results make a parallel to the age effects on piglet calls after weaning [17]. However, the type of the situation, its duration and the age variation is quite different in the two studies. In spite of the differences, both studies show that piglets at different stages of early ontogeny adjust their level of signalling to the degree in which they need maternal intervention into their fitness-threatening situation. 

The notion that the variability in S-calls reflects the needs of the piglet was also supported by the finding that lighter piglets emitted more calls than heavier piglets, in agreement with Prediction 1. The rate of calling is a prominent vocal signal and may be sufficient for the sows to react more strongly when a vulnerable small piglet is trapped under her body. However, none of the acoustic parameters of S-calls was affected by body weight, showing that the effect of weight on S-calling was less pronounced than the effect of age. This might indicate that the more mature locomotory skills of week-old piglets are more important in the prevention of crushing than the body weight itself. 

### The squeezed calls – health effects

Differences in vocal expression between healthy and diseased individuals have been recorded in several species [4,40,41]. We predicted (Prediction 2) that sick piglets would vocalize more intensely, based on the assumption that compromised health is another example of vulnerable condition (i.e. of a high level of need), in addition to young age and low weight. However, piglet health status did not affect the intensity of calling during the squeezed situation in our study. One reason for this negative finding might be that the health problems of our piglets were not serious enough. The health status of the experimental litters was generally good, as reflected by the fact that none of the sick piglets suffered from more than one health problem and that there were not enough sick piglets to test the health effects for both the S-calls and the I-calls. However, we cannot exclude the possibility that health status is reflected in other aspects of vocal quality that were not measured in the current study.

### The isolation calls

Vocalizations by isolated piglets were not affected by either age or body weight. Thus the Prediction 3 that the greater need for maternal contact by the one-day old piglets and by the lighter piglets would be communicated in more intensive vocalizations was not supported. This is in contrast to the previously reported associations of both age [17] and body weight [14] with distress vocalization intensity during experimental isolation of piglets. The lack of body weight effect in our study is especially surprising since Weary and Fraser [14] used piglets that were not much older (10 days), had similar distribution of body weights and were tested in a similar way. One difference in the experimental procedure was that in the current experiment, the piglets were isolated immediately after a sucking bout (i.e. fully satiated with milk) whereas Weary and Fraser [14] took the piglets away “while they were sleeping” so presumably somewhere between two nursings. Thus our piglets were generally less in need, which might have obscured any potential influence of body weight on calling. Weary and Fraser [14] noted that there was a tri-modal distribution of the calls’ acoustic frequencies, and that the differences between heavy and light piglets in their study were only apparent in the category of the highest calls, i.e. those with the loudest frequency band greater than 500 Hz. In the current study, the calls had generally lower pitch than in the study by Weary and Fraser. For instance, the PF mean parameter equaled 455 Hz and 398 Hz, respectively, at the 2-4 and 8-10 minute of the isolation in our study, whereas the graphs in Weary and Fraser [14] indicate values of 750 Hz and 550 Hz at the corresponding intervals. Thus it is possible that the more high-pitched calls for which Weary and Fraser [14] found body weight influence were rare in the current study in which satiated piglets were used.

More generally, the calls emitted by piglets in a short-term separation from the mother may be of different types [19] and fulfill various functions. The lower-pitched calls that piglets mostly emitted in our study may in fact be contact calls rather than full-scale distress calls. Indeed, experiments have shown that the calls of isolated piglets stimulate sows to vocalize back [15,21,25] and vice versa, so the primary role of these lower-pitched calls may be to get a vocal response and to locate the mother. It is possible that only the higher-pitched calls convey the message about the need of the piglet and may therefore increase when a more distressful situation occurs such as a combination of low weight and being hungry. Indeed, very high pitch calls with a mean frequency over 1000 Hz were found by [15] in low-weight and hungry piglets isolated in cold environment.

In contrast to the squeezed calls, no age effect was found in the isolation calls. If the isolation calls are indeed mainly contact calls with the function of eliciting a vocal response from the sow, then the propensity to emit these calls may be similarly important for both 1 day old and 7 day old piglets.

In summary, the squeezed vocalizations were much more intense on Day 1 than on Day 7, probably reflecting the much higher danger for neonatal piglets of being fatally trapped under the body of the mother sow at this time. Lighter piglets also called more in the squeezed situation. In these two respects, the model of honest signalling of need was confirmed. No difference between the squeezed calls of the sick and the healthy piglets was found. The isolation calls were not affected by either the body weight or by the age of the piglets, possibly because the levels of distress were low and calls served to establish vocal response by the sow rather than to communicate the level of need. We conclude that the model of honest signalling of need applies to some, but not all cases of piglet-to-sow vocal communication.
